# Chlorophyll *a* Fluorescence in Evaluation of the Effect of Heavy Metal Soil Contamination on Perennial Grasses

**DOI:** 10.1371/journal.pone.0091475

**Published:** 2014-03-14

**Authors:** Grzegorz Żurek, Krystyna Rybka, Marta Pogrzeba, Jacek Krzyżak, Kamil Prokopiuk

**Affiliations:** 1 Department of Grasses, Legumes and Energy Plants, Plant Breeding and Acclimatization Institute IHAR-PIB, Radzików, Błonie, Poland; 2 Department of Plant Physiology and Biochemistry, Plant Breeding and Acclimatization Institute IHAR-PIB, Radzików, Błonie, Poland; 3 Department of Environmental Biotechnology, Phytoremediation Team, Institute for Ecology of Industrial Areas, Katowice, Poland; University of Hyderabad, India

## Abstract

Chlorophyll *a* fluorescence gives information about the plant physiological status due to its coupling to the photosynthetic electron transfer chain and to the further biochemical processes. Environmental stresses, which acts synergistically, disturbs the photosynthesis. The OJIP test, elaborated by Strasser and co-workers, enables comparison of the physiological status of plants grown on polluted vs. control areas. The paper shows that the Chl *a* measurements are very useful tool in evaluating of heavy metal ions influence on perennial grasses, tested as potential phytoremediators. Among 5 cultivars tested, the highest concentration of Cd and Zn ions, not associated with the yield reduction, was detected in the biomass of tall fescue cv. Rahela. Chl *a* fluorescence interpreted as double normalized curves pointed out Rahela as the outstanding cultivar under the HM ions stress.

## Introduction

Phytoremediation requires plants that maintain good fitness on contaminated soils in parallel with the highest possible concentration of pollutants in aerial parts [1]. For evaluation of species and varieties as potential phytoremediators in breeding programs, low-cost, easy to carry and high throughput techniques are required. Chlorophyll *a* (Chl *a*) fluorescence measurements fulfill those conditions and in parallel with detection of heavy metal (HM) ions concentration represent a good screening tool for evaluation of plant suitability to grow on polluted lands.

Chl *a* fluorescence is a natural phenomenon, characteristic for all photosynthetic organisms, since the sun light energy absorbed by chlorophyll molecules, if not used to drive photosynthesis, is dissipated as heat radiation or is re-emitted as light photons. Even though the amount of fluorescence usually makes no more than 1–8% of total light absorbed, the fact that light induced processes are as communicating vessels and the increase in the efficiency of one of them results in the decrease in the yield of the other two, measurement of Chl *a* fluorescence gives information about changes in the efficiency of photochemistry and heat scattering [2], [3]. Since such a measurement is convenient, non-invasive, highly sensitive, rapid, reliable and gives quantitative probe of oxygenic photosynthesis, it is in common use as suitable tool for determination of plant physiological status [4]. It can be read in the way as the electrocardiograms are (Goltsev, personal communication, 2012). Plants subjected to HM ions stress show disturbances in photosynthesis due to single or cumulative phenomena of: *i/*direct interaction of HM ions with protein thioyl-, histidyl- and carboxyl-groups; *ii/*formation of reactive oxygen species (ROS); and/or *iii/*displacement of essential cations in protein active centers. Some ions such as Hg, Cu, Cd, Ni or Zn may substitute the central Mn ion in chlorophyll molecule, forming chlorophyll-metal complexes and thus lower PSII quantum efficiency [5], [6], [7]. These circumstances affect most of detected parameters [8]. Beside the comparison of particular parameters, among which the F_V_/F_0_ and F_V_/F_m_ are the most widely known and used, the interpretation of double normalized curves, based on JIP test is an increasingly popular approach in applied, environmental studies [9]. Plots are drown from the data recorded with high sampling rate within a second after the exposure of dark adapted leaf to the light, on logarithmic timeline as the independent variable [10]. On such graphs points of inflections (J-I-P), noted on the increasing part of the registered fluorescence, are the base of inference about the structure and function of the photosynthetic apparatus, with the obligatory assumption, that the efficiency of chlorophyll antenna in light energy capture is the only cause of detected fluctuations and that the only limitation of the photochemical conversion in PSII, is plastoquinone Q_A_, the electron acceptor [11], [12]. Even though the accurate interpretations of Chl *a* fluorescence measurements, which are the result of in-depth knowledge accumulated over the years, still needs the further studies [13] both: the knowledge and the modern technology have turned measurements of Chl *a* fluorescence into convenient and reliable tool for outdoor, environmental studies [9].

In the field studies, the experimental system is complex. All environmental stimuli: contaminants in parallel with weather fluctuations act synergistically, so Chl *a* fluorescence reflects theirs cumulative effect. For phytoremediation - revealing plants of better physiological activity on contaminated soils, in parallel with higher concentration of HM ions in aerial parts, is the goal. However it should be considered that good physiological state of the plant detected in contaminated rural areas can be misleading, since health risk for organisms in the food chain. The phytotoxicity of some metals has been long established [14].

The aim of our research was to evaluate the usefulness of Chl *a* fluorescence as a method for phytoremediation studies in field experiments on five perennial grass species grown on naturally HM contaminated soils. The acquired knowledge is to be used in planning of biomass processing systems and, in parallel, in phytoremediation of polluted soils, using cultivation of energy plants as a main concept.

## Materials and Methods

### Field Experiment

The experiment was located on contaminated soil on Silesia, southern Poland (50.4^o^N/018.9^o^E). Plots were established in the vicinity of a closed-down cadmium/lead/zinc/ore mine and processing plant, which operated for more than 100 years and had significant impact on local soils. Reference site (uncontaminated soil) was located in a distance of 25 km North from above, (50.6^o^N/018.88^o^E) on field with similar forecrop history. Permission was not required for research activities on both locations. No endangered or protected species were involved in our field studies.

### Plant Materials

Five perennial grass cultivars: tall oat grass, *Arrhentherum elatius* L. (Ae.) ‘Wiwena’; rescuegrass, *Bromus carinathus* Hooker et Arn (Bc.) ‘Broma’; tall wheat grass, *Elytrigia elongata* Nevski (Ee.) ‘Bamar’; smooth brome grass, *Bromus inermis* Leyss (Bi.) ‘Brudzynska’ and tall fescue, *Festuca arundinacea* Schreb. (Fa.) ‘Rahela’ were sown at the end of June 2010 on 1×1 m plots, in 3 replications per variety on both locations. No further treatments such as: fertilization, herbicides or watering were applied, despite of collecting aboveground biomass at the end of growing seasons in 2010 and 2011. All observations and measurements: plant height [PH, cm], generative shoots abundance [GS, %] per plot area and yield [BY, t·ha^−1^] as well as Chl *a* fluorescence were conducted at the first year after the sowing (2011) at both locations: all parameters were measured in June, beside the biomass yield which was measured in October.

### Soil Analysis

Five representative soil samples were collected from 0–30 cm horizon at each site for chemical analysis. Large chunks of soil were spread out to dry in a forced air oven at 35°C and then were grounded to pass a 60 mesh screen.

Soil pH was measured in 1 M KCl and H_2_O (1∶2.5) suspension (combination glass/calomel electrode and pH meter) and electrical conductivity (EC) e in water extract (1∶2.5) after 24 h of equilibration; the fraction of bioavailable metals was obtained by extraction of 3 g of air-dried soil (ground to <0.25 mm) with 30 ml 0.01 M CaCl_2_ for 5 h; total metal concentrations by extraction of air-dried soil ground to <0.25 mm with concentrated HClO_4_ and HF.

Concentrations of metals in soil extracts were detected using inductive coupled plasma spectrometry (ICP-AES) (Spectro Analytical Instruments GmbH, Kleve, Germany).

### Determination of HM Ions Concentration in Plant Biomass

Plant material was washed with tap water and then with deionised water in an ultrasonic washer to remove all soil particles followed by drying at 70^o^C for 3 days. One g of dried, ground material was wet-ashed using concentrated nitric acid (Merck) in a microwave system (MDS 2000, CEM, USA). Concentration of lead, cadmium and zinc ions in plant material was also detected using inductive coupled plasma spectrometry (ICP-AES).

### Measurements of Chl *a* Fluorescence Transient

Chl *a* fluorescence was measured using PocketPEA portable fluorometers (Hansatech Instruments, King’s Lynn, Norfolk, UK). Recordings were performed simultaneously on both locations at 10–12 a.m. UMT on the first fully developed leaf, (3 leaves per 5 plants per replication). Dark adaptation (0.5 hour) of the middle part of abaxial leaf blade preceded the measurement. Fluorescence was induced by saturating, red actinic light with energy of 3.500 µmol m^−2^ s^−1^, and first 3 seconds of transient fluorescence, covering more than its exponential growing part, was registered with time intervals increasing from 10 µs within first 300 µs of the measurement up to 100 ms intervals for times longer than 0.3 sec. Additionally, collected data points were double normalized between points O-I, O-J, O-K and a kinetic differences ΔW_OI_, ΔW_OJ_ and ΔW_OK_ were calculated and analyzed as graphs on logarithmic time scale [10].

List of measured parameters:

**F_O_≈F_50 µs_** [minimal fluorescence];

**F_1_**, **F_2_**, **F_3_**, **F_4_**, **F_5_** [fluorescence at times: 0.05, 01, 03, 2.0 and 30 ms after the start of actinic illumination F_O_. Values at 0.3, 2.0 and 30 ms responds to fluorescence at K, J, I points of inflections, on fluorescence transient curve];

**F_M_ = F_P_** [maximal recorded fluorescence];

**t_FM_** [time (in ms) to reach the maximal fluorescence, F_M_];

**Area** [total complementary area between the fluorescence induction curve and F_M_ of OJIP curve].

Calculated parameters:

a) listed by PocketPEA software:

**F_V_** [maximal variable fluorescence calculated as F_M_–F_O_];

**F_V_/F_M_** [force of the light reactions];

**RC/ABS** [the amount of active reaction centers per absorption];

**(1–V_J_)/V_J_** [measure of forward electron transport];

**PI_ABS_** [performance index];

b) not listed by PocketPEA software:

**W_OI_, W_OJ_** and **W_OK_** double normalized fluorescence readings at points O-I, O-J, O-K [ratio of variable fluorescence (F_t_−F_O_) to the amplitude: (F_I_−F_O_), (F_J_−F_O_) or (F_K_−F_O_)];

**ΔW_OI_, ΔW_OJ_** and **ΔW_OK_** [difference of: [W_OI_(reference)−W_OI_(control)], [W_OJ_(reference)−W_OJ_(control)] and [W_OK_(reference)−W_OK_(control)], respectively]. Calculated separately for each cultivar.

### Climatic Data

Monthly precipitation and air temperatures for both localities were estimated on the basis of free climatic service (http://klimat.icm.edu.pl/serv_climate.php), where air temperature and precipitation data for current and past periods can be projected for given geographic coordinates in Poland (Table 1).

**Table 1:** Monthly precipitation and mean temperatures during the course of experiment in polluted and reference site.

Year	Month	Precipitation (mm)		Temperature (°C)	
		Site type:			
		polluted	reference	polluted	reference
2010	1	43.4	40.3	−6.2	−6.6
	2	28.0	25.2	−1.3	−1.5
	3	31.0	31.0	3.8	3.5
	4	45.0	39.0	8.9	8.8
	5	232.5	217.0	12.8	12.6
	6	66.0	60.0	17.0	16.8
	7	108.5	108.5	20.4	20.3
	8	102.3	102.3	18.6	18.6
	9	99.0	87.0	12.5	12.4
	10	6.2	6.8	6.3	6.2
	11	69.0	72.0	6.5	6.2
	12	49.6	46.5	−5.2	−5.5
2011	1	28.2	27.9	−0.6	−0.6
	2	12.0	11.2	−2.2	−2.5
	3	37.5	31.0	4.1	4.0
	4	24.3	21.0	10.7	10.7
	5	72.5	62.0	13.6	13.6
	6	51.3	54.0	18.3	18.1

### Statistical Calculations

Statistical calculations were performed using SAS statistical package. The least significant differences (LSD) between means were calculated according to the Duncan honesty test and significance of difference between means was tested with the probability of 95%. Divergence among tested cultivars was estimated using Principal Component Analysis (PCA) analysis, based on correlation matrix algorithm for Chl *a* fluorescence traits.

## Results

In order to examine, to what extent the Chl *a* fluorescence was useful in studies of the effect of HM polluted soil in natural agro-ecosystem, localizations with the most similar soil composition (beside HM ions concentration) were chosen to run the experiment. Soils did not differ much in organic matter content: 4% on contaminated area vs. 3.5% on reference place. The pH of contaminated soil was nearly neutral (6.8), whereas the reference one was slightly acidic (5.2). Although the content of silt and clay were on similar level, the reference soil was 1.6 times more sandy than contaminated soil. The amount of HM ions in polluted soil exceeded by almost 2 orders of magnitude the concentration of Cd and Zn ions and about 50 times of Pb ions when compared to reference place (Table 2).

**Table 2:** The physico-chemical characteristics of the contaminated and the reference soil, where, under the natural conditions, the tested plants were cultivated.

Soil property	Soil type:	
	contaminated	reference
pH (KCl)	6.79±0.01	5.17±0.02
pH (H_2_O)	7.01±0.03	5.96±0.02
EC (µS cm^−1^)	98.23±7.01	63.90±9.59
organic matter content (%)	4.0±0.03	3.5±0.03
*Soil composition*		
clay (<0.002 mm). %	16.1±2.0	12.7±1.5
silt (0.002–0.05 mm). %	55.9±3.1	40.0±2.2
sand (0.05–1 mm). %	28.0±2.0	47.3±1.1
*Total heavy metal concentration (extraction with aqua regia)*		
Pb (mg kg^−1^)	547.0±27.92	16.76±0.50
Cd (mg kg^−1^)	20.84±1.17	0.74±0.04
Zn (mg kg^−1^)	2174±103.0	35.06±0.69
*CaCl_2_ extractable metal fraction*		
lead [Pb^+2^] (mg kg^−1^)	16.76±0.50	0.019±0.01
cadmium [Cd^+2^] (mg kg^−1^)	0.74±0.04	0.016±0.01
zinc [Zn^+2^] (mg kg^−1^)	35.06±0.70	0.533±0.02

Climatic data for both localities were also very similar (Table 1). Reference site received similar amount of rain and heat during vegetation in 2010 as well as in 2011.

It is very likely that the elevated concentration of Pb, Cd and Zn ions in the soil affected plant growth (Table 3). Biomass yields of plants grown on the polluted site ranged from 1.7 (cv. Broma) to 3.7 (cv. Wiwena & Rahela) t·ha^−1^ of dry mass and it was ca. 0.8 t·ha^−1^ lower than the yield of grasses grown on the reference site. The highest reduction of the yield were noted for brome grasses: cv. Brudzynska (2.2 t·ha^−1^) and cv. Broma (1.1 t·ha^−1^), grown on polluted site. High biomass yields of cv. Wiewena and cv. Rahela were associated with the significantly lower GS value, as compared to the reference site. No significant differences in GS values were detected for other cultivars grown on both sites. Cultivar Bamar grown on the polluted soil did not differ significantly from that grown on the reference one, beside minor disparity in the Chl *a* fluorescence parameters.

**Table 3:** The agronomic characteristics of grass cultivars grown on the contaminated agricultural soil in comparison to the plants cultivated on the reference soil: yield of the biomass (BY) [t ha^−1^] and abundance of generative stems (GS) [%].

Grass cultivar	Trait	Site type:		Significance of difference
		polluted	reference	
(Ae.) Wiwena	BY [t ha^−1^]	3.7±0.1	4.3±0.7	ns.
	GS [%]	8.3±0.2	18.3±0.9	***
(Ee.) Bamar	BY [t ha^−1^]	2.0±0.1	1.7±0.2	ns.
	GS [%]	1.0±0.1	1.3±0.2	ns.
(Bc.) Broma	BY [t ha^−1^]	1.7±0.2	2.8±0.9	***
	GS [%]	86.7±8.1	90.0±7.1	ns.
(Bu.) Brudzynska	BY [t ha^−1^]	1.8±0.2	4.0±0.9	***
	GS [%]	15.0±1.1	16.7±2.1	ns.
(Fa.) Rahela	BY [t ha^−1^]	3.7±0.9	4.3±1.0	ns.
	GS [%]	9.0±0.8	26.7±3.7	**

Plant height (PH) [cm] values are not shown since it did not differentiate studied cultivars. ns – not significant; **significant for P>95%; ***significant for P>99%.

The differences in HM ions sequestration were detected as well (Table 4). The highest concentrations of Cd and Zn ions were found in the biomass of cv. Rahela whereas the lowest concentration of Cd was detected in cv. Bamar and Broma. The least Zn ions were collected by the cv. Wiwena. Rahela accumulated 3.9 fold more of Cd ions and 2.2 more of Zn ions. No statistically important differences in Pb ions accumulation were noted.

**Table 4:** The concentration of HM in plant biomass.

Grass cultivar	Soil type	HM concentration [mg kg^−1^]		
		Cd	Pb	Zn
(Ae.) Wiwena	reference	0.11±0.02	0.31±0.08	20.45±1.0
	polluted	3.0±0.1	64.0±4.5	185.5±6.4
(Ee.) Bamar	reference	0.14±0.01	0.25±0.01	18.45±2.1
	polluted	2.2±0.2	31.0±4.2	253.0±2.8
(Bc.) Broma	reference	0.08±0.01	0.35±0.02	16.7±1.8
	polluted	2.2±0.6	51.0±9.9	197.0±43.8
(Bu.) Brudzynska	reference	0.18±0.03	0.37±0.02	25.25±5.4
	polluted	3.5±0.4	59.5±12.0	256.0±4.2
(Fa.) Rahela	reference	0.19±0.02	0.73±0.1	19.5±2.0
	polluted	8.5±0.9	50.0±11.3	417.0±50.2
LSD (P>95%)	reference	n.s.	n.s.	n.s.
	polluted	1.33	n.s.	113.5

n.s. – not significant.

Inhibition of the plant growth as a reaction on HM ions stress was the result of disturbances in the plant cell metabolism affecting also the chloroplasts and reflected in changes of the fluorescence parameters (Table 5). Fluorescence intensities at different time points distinguished cultivars. At points F_M_, F_V_ and F_5_ the recorded data were the smallest for cv. Rahela, whereas for shorter times (from F_1_ to F_4_) cv. Bamar. was characterized by the weakest signal. Also the F_V_/F_M_ ratio as well as the other calculated parameters were the lowest for cv. Rahela.

**Table 5:** The parameters of Chl *a* fluorescence of the grass cultivars grown on the polluted and the reference soil.

Chl *a* fluorescenceparameters	Soil type	Grass cultivars				
		(Ae.) Wiwena	(Ee.) Bamar	(Bu.) Brudz.	(Bc.) Broma	(Fa.) Rahela
*measured parameters*:						
F_1_ = F_50µs_ = F_O_(AU×10^3^]	reference	6.9	6.9	8.0	7.2	7.2
	polluted	5.0 ab	4.6 b	4.9 ab	5.3 ab	5.5 a
	sign. of diff.	**	*	***	**	***
F_2_(AU×10^3^]	reference	7.7	7.3	8.7	7.9	8.0
	polluted	5.4 ab	5.0 b	5.4 ab	5.8 ab	6.2 a
	sign. of diff.	***	*	***	**	**
F_3_(AU×10^3^]	reference	10.6 ab	9.1 b	12.1 a	11.0 ab	11.2 ab
	polluted	7.2 bc	6.4 c	8.0 ab	8.4 ab	8.7 a
	sign. of diff.	***	*	**	**	**
F_4_(AU×10^3^]	reference	16.7 ab	14.3 b	19.9 a	17.2 ab	17.7 ab
	polluted	13.3 b	11.5 c	14.0 ab	15.0 a	13.6 ab
	sign. of diff.	**	n.s.	**	***	***
F_5_(AU×10^3^]	reference	25.7	26.1	28.6	26.5	25.7
	polluted	20.1 ab	18.7 ab	19.2 ab	20.4 a	18.3 b
	sign. of diff.	**	**	***	**	***
F_M_[AU×10^3^]	reference	31.7	33.9	34.5	31.6	30.5
	polluted	24.8 a	22.5 ab	22.4 ab	24.0 a	20.2 b
	sign. of diff.	**	**	***	**	***
F_V_[AU×10^3^]	reference	25.5	27.5	27.4	25.2	24.1
	polluted	20.3 a	18.3 ab	18.2 ab	19.3 ab	15.3 c
	sign. of diff.	**	***	***	***	***
Tfm	reference	750 a	733 a	550 ab	385 b	550 ab
	polluted	500	650	450	500	850
	sign. of diff.	****	n.s.	n.s.	n.s.	n.s.
Area [AUx10^3^]	reference	977 b	1223 a	930 bc	652 d	721 cd
	polluted	651 a	551 ab	455 b	433 bc	280 c
	sign. of diff.	**	***	***	*	***
*calculated paramaters:*						
F_V_/F_M_	reference	0.806	0.812	0.794	0.798	0.791
	polluted	0.818 a	0.811 ab	0.811 ab	0.804 b	0.760 c
	sign. of diff.	**	n.s.	**	n.s.	***
RC/ABS	reference	1.15 b	2.31 a	1.12 b	1.1025 b	0.985 b
	polluted	1.53 a	1.72 a	0.98 b	1.058 b	0.761 c
	sign. of diff.	**	n.s.	n.s.	n.s.	*
PI	reference	2.80 b	7.38 a	2.32 b	2.48 b	1.97 b
	polluted	3.90 b	4.46 a	1.94 c	2.03 c	1.04 d
	sign. of diff.	**	n.s.	n.s.	*	***
(1–Vj)/Vj	reference	0.59 b	0.72 a	0.53 b	0.57 b	0.53 b
	polluted	0.57 b	0.61 a	0.46 cd	0.47 c	0.43 d
	sign. of diff.	n.s.	**	*	**	***

The cultivars were compared within soil types (reference and polluted) by the LSD test (^1^) as well as the influence of the soil type on the cultivar was tested by LSD test (^2^). Values with the same small letters are not different significantly within soil type (rows). *- p>95%; **− P>99%; ***- P>99.9%; n.s. - not significant.

**Figure 1:**
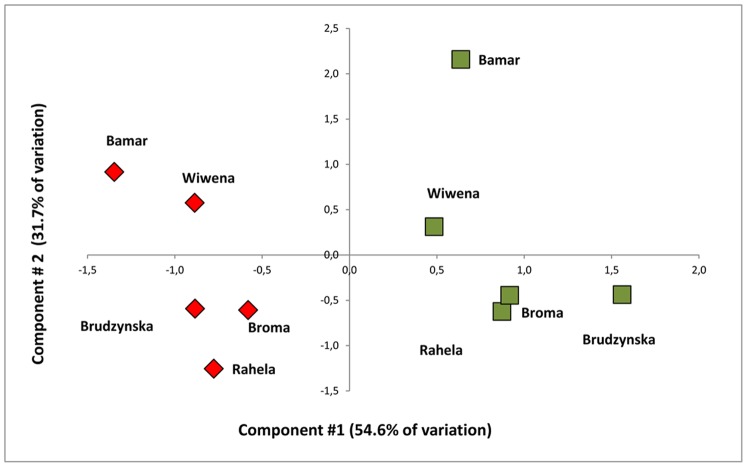
The distribution of tested cultivars in PCA plot created on the basis of Chl *a* fluorescence data. The first two components, accounted about 86% of total variation: 1^st^ was related to measured values (FM, FV, F1-F5), 2^nd^ was highly correlated with calculated parameters (RC/ABS, (1-Vj)/Vj and PI. Cultivars tested in unpolluted site- green boxes; polluted site- red boxes.

Principal Component Analysis (PCA) enabled reduction in dimension of multivariate fluorescence data set. The first two components, accounted about 86% of total variation: 1^st^ –54.6% of total variation explained, and was highly correlated with measured values: F_O_, F_M_, F_V_, F_1_–F_5_; 2^nd^ explaining 31.7% of total variation, was highly correlated with parameters: RC/ABS, (1–V_j_)/V_j_ and P_I_ (Table 6). The multi-trait analysis visually presents the overall magnitude of changes related to the plant growth in polluted and unpolluted site across all Chl *a* parameters (Fig. 1). Cultivars tested in unpolluted *vs.* polluted site could be easily distinguished on the basis of location on *x*-axis (measured parameters of Chl *a* fluorescence).

**Table 6:** Eigenvector values of three components calculated after Principal Component Analysis (PCA) performed on Chl *a* fluorescence parameters.

Chl *a* fluorescence parameters	Component number:		
	#1	#2	#3
F_O_	0.98	0.13	−0.13
F_M_	0.92	0.39	0.09
F_V_	0.88	0.45	0.14
F_V_/F_M_	−0.16	0.66	0.71
T_FM_	−0.11	0.18	−0.94
AREA	0.66	0.70	−0.06
F_1_	0.99	0.03	−0.13
F_2_	0.99	−0.04	−0.14
F_3_	0.96	−0.27	−0.07
F_4_	0.93	−0.30	0.15
F_5_	0.97	0.21	0.07
RC/ABS	−0.04	0.97	0.04
V_J_	0.30	0.93	−0.02
P_I_	0.02	0.98	0.01
Eigen values	8.19	4.75	1.71
Variaton explained (%)	54.6	31.7	11.4

**Figure 2:**
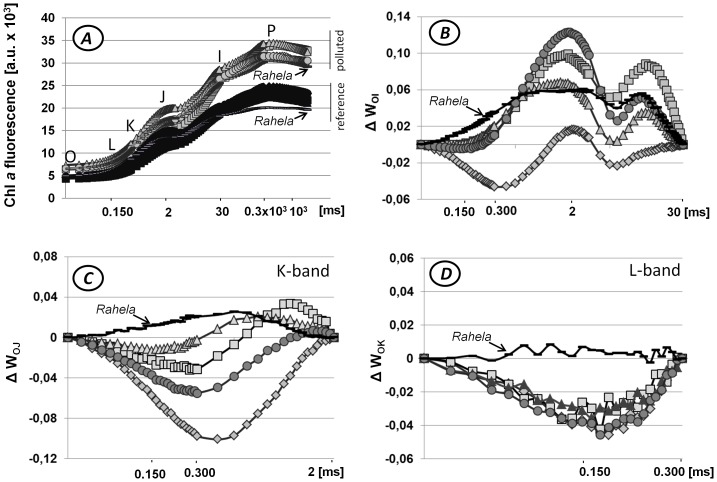
The chlorophyll a fluorescence transients (OJIP) from dark adapted leaves of five perennial grass cultivars given on a logarithmic time scale from 50 µs to 1 s. **A/** The fluorescence registered for cultivars grown on the reference and the HM ions polluted soil plotted on a logarithmic time scale. The time points for the calculation of structural and functional parameters of the JIP test are marked: O – the fluorescence intensity at 50 µs, L – at 150 µs, K – at 300 µs, J – at 2 ms and I – at 30 ms, P – the maximal fluorescence intensity at the time about 1 s, denoted as t_FM_; **B/**ΔW_OI_ – the difference of double normalized data at points F_O_ and F_I_ for each cultivar grown on polluted and reference soil where W_OI_ = [(F_t_−F_O_)/(F_I_−F_O_)]; bands in the time range 0.050–30 ms; **C/**ΔW_OJ_ – the difference of double normalized data at points F_O_ and F_J_ for each cultivar grown on polluted and reference soil where W_OJ_ = [(F_t_−F_O_)/(F_J_−F_O_)]; K band; D/ΔW_OK_ – the difference of double normalized data at points F_O_ and F_K_ for each cultivar grown on polluted and reference soil where W_OK_ = [(F_t_−F_O_)/(F_K_−F_O_)]; L-band. Cultivars are marked as follow: diamonds – (Ae.) ‘Wiwena’; squares – (Ee.) ‘Bamar’; triangles – (Bi.) ‘Brudzynska’; circles – (Bc.) ‘Broma’; black dashes – (Fa.) ‘Rahela’.

Splits of the cultivars into groups on PCA diagrams induced the idea for calculation of mean values of fluorescence data points for each of plant group: those from polluted soil and those from reference one, in order to present the average values on logarithmic time scale. The fluorescence transient of plants grown on HM ion contaminated soil were lower than those of plants grown on the reference site. Points of inflection: J at 2.0 ms and I at 30 ms, important for the results interpretation, are visible there (Fig. 2a). Other points of local inflections: L at 150 µs, K at 300 µs, identified and described by Strasser [15] are so tiny that can be visible as local extreme points only after additional mathematical operations; here, the differences of double normalized transients measured for plants grown on polluted and reference sides are presented, as described in Materials and Methods. Double normalization of the data at points O and I, reflects all local disturbances originating from the physical and biochemical events occurring along the electron transport chain (ETC) in chloroplasts: from the electron trapping through the plastoquinone reduction, and further towards the sink at PSI acceptor side [15]. Curves drown for all cultivars, but not cv. Wiwena, have positive values (Fig. 2b). Point of inflection J, at about 2 ms, is detectable for all cultivars. Point K, at about 0.3 ms, is sharp for cv. Wiwena. Point L, at 0.150 ms, is not visible on the Fig. 2b. For all cultivars extremes not discussed by Tsimilli-Michael and Strasser [15] at about 5 ms (the minimum) and at about 10 ms (the maximum) are visible. Shape of the curve drown for cv. Rahela is noticeably different, more flat, then curves of the other ones.

Normalization procedure at points O and J, and differentiation of results from polluted and reference soils, visualized the K-band, the most expressed with minus sign, for cv. Wiwena and the weakest for cultivars: Brudzynska (with the negative sign) and Rahela (with positive one) (Fig. 2c). Normalization at points O and K visualized L-band (Fig. 2d). All curves, but not that drawn for cv. Rahela, turned out to be almost identical, with the minus sign. The plot for the cv. Rahela was close to zero.

## Discussion

The detected differences in soil composition of contaminated and reference site resulted from outdoor natural environments of rural areas, where the experiments were run. The fact that the vegetation seasons prior to Chl *a* fluorescence measurements were rainy and water availability did not limited the plant growth, opened the opportunity to interpret the differences in grasses performance as the response to HM. More sand in soil at reference site might negatively affect the plant growth and performance but mostly during the low precipitation periods. In March, April and May, three months prior to Chl *a* measurements, polluted and reference site received 107% and 91% of rainfall long term average (1971–2000), respectively, which periods are defined as to average precipitation periods in Poland [16]. Lower EC and pH at reference site could be the indirect effect of lower level of HM concentration, so it was assumed, that detected differences in plant performance resulted mostly from high HM level in soil. The HM ion concentration in polluted site exceeded four fold the limits of Cd and Pb ions concentration for arable soils and six fold the limits of Zn, which are: 12.7 mg·kg^−1^ Cd, 304.5 mg·kg^−1^ Pb, and of 1677.5 mg·kg^−1^ Zn (Table 2). Plants can tolerate relatively large amount of zinc in the soil, because it is an essential micronutrient, which deficiency as well as excess is harmful [17].

Contamination of agricultural land by trace metals is the side effect of industrialization. In regions of long history of industrial emission elevated levels of cadmium, lead, zinc and other ions emitted in the course of coal and ore mining and processing are detected. Since the maintenance of agricultural areas in those regions is important from ecological and sociological standpoint, the alternative farming activities are needed. Perennial grass biomass production for energy purposes is one of such solution [18], [19]. However, caution should be made on practical use of biomass with high amounts of heavy metals. Considering its chemical composition it is not a substrate for direct energy production but a hazardous waste. Contaminated crop disposal process must be always planned in any phytoremediation activities, especially when large quantities of biomass are to be generated [20].

Many plant species has been already screened for their ability to accumulate HM. For this purpose two strategies have been tested: *i/*the cultivation of hyperaccumulators on polluted sides (usually plants of relatively low aboveground biomass, accumulating high amounts of one or more toxic ions); *ii/*growing plants which produce high biomass, despite lower ability of HM sequestration [21]. The latter seems much promising, because there are numbers of species available from many families, from which willows and perennial grasses are the most interesting [22]. Although the willow produces the high biomass with the effective nutrient uptake as well as the clone specific capacity for HM uptake, high demand for water and the specific harvest equipment required limit its usefulness to the large and flat fields rich in water, what is not frequent in the case of polluted areas [23]. Considering perennial grasses, there are no such limitations. A number of perennial grasses are used increasingly in Europe and North America as renewable bioenergy sources, as they can be grown with low maintenance on marginal soils and harvested to produce large volumes of biomass [24].

Dynamics of HM ions absorption, transport and neutralization influence theirs toxicity. It was shown, that dose dependent relative cadmium toxicity is 4-fold higher than zinc and 3-fold higher than lead, for the onion epidermal cells used as a model. The way of HM inactivation affects theirs toxicity: HM ions can be stored in vacuoles, Cd ions can form the complexes with the cell wall polymers, Pb can be locked in endoplasmic reticulum vesicles [25]. Non neutralized HM ions induce formation of an excess of free radicals and slowdown turnover of chloroplasts proteins, mostly D1 protein [17], [26]. Chloroplasts development in young leaves as well as chlorophyll and β-carotene content and Chl *a*/*b* ratio is also affected by Cd ions. Zinc and cobalt divalent cations have the ability to replace Mn^+2^ or Fe^+2 ^in chloroplast proteins active centers, decreasing theirs functional activity [27], [28].

Thus the HM impact on the plant interferes with photosynthesis and the measurement of Chl *a* fluorescence reveals the disturbances. At the beginning, the fluorescence of dark-adapted leaf rises rapidly from the minimal level (O), typically recorded at 50 microsecond, to the maximum (marked as ‘M’ or ‘P’), usually appearing at about one second. Minimal fluorescence reflects the oxidized state of quinone Q_A_, whereas the maximal one – the completely reduced state of the whole plastoquinone pool (PQ) [15]. This increasing part of the fluorescence transient shows the dynamics of light induced electron flow through the electron transfer chain (ETC) in chloroplasts: pheophytin → plastoquinone pool (PQ) → cytochrome b6f complex (cyt-bf) → plastocyanin (PC) up to the end electron acceptors on PSI acceptor side. ETC damage and sink dependent slowdown of electron flow are visible directly on fluorescence curves drawn on logarithmic time abscissa or on double normalized curves as well as can be recognized by parameters calculated using JIP-test and its applications [3].

Fluorescence values F_2_, F_3_, F_4_ and F_5_ at times: 0.15, 0.30, 2.0 and 30 ms responds to L, K, J, I points of inflections, on fluorescence transient curve. Usually under the stress conditions the fluorescence transient curve becomes flatter, i.e.: the F_M_ decreases [29]; almost all Chl *a* fluorescence induction parameters (F_o_, F_v_, F_m_) were found to be affected by HM concentration in different plant species [8]. The curve deformation depends on both: the resistance of the plant as well as on the time of exposure to the stress; in outdoor studies- to stresses/environmental conditions acting synergistically. The change of F_M_ entails changes of the other F’s, which is well documented and as a consequence affects the values of calculated parameters [30], [31].

Our data distinguished cv. Rahela as the cultivar which reached the highest HM ions concentration in aerial parts and its photosynthesis was the most influenced by them. The maximum quantum yield of PSII photochemical reaction described by parameter *F*v/*F*m measured on the reference area was slightly lower than respected for the healthy plants (0.83 vs. 0.79) and decreased upon reference side conditions (0.76). It indicated a decline of the maximum efficiency at which light absorbed by plants grown on the polluted soil is used for the reduction of Q_A_ [12], [27]. Other cultivars did not show differences of F_v_/F_m_ parameter, which can happen [32] and can be explained by much lower concentration of sequestrated HM ions. Kalaji *et al.* [12] discussed the dynamics of J-I-P parameters reflecting the stress resistance: the time to reach F_M_ value (T_FM_), reduced plastoquinone pool size (Area) and the vitality index of PSII (PI) were more sensitive for action of the stress than commonly used parameters of maximal quantum yield of PSII: F_V_/F_M_ and F_V_/F_O_.

In our studies the first component of PCA analysis depended on measured values: F_M_, F_V_, F_1_–F_5_, while the second on calculated parameters: RC/ABS, (1–Vj)/Vj and PI. Grouping of cultivars calculated on bases of 2 principal components which explained 85% of differentiation, gave logical results. Cultivars were grouped in sub-classes as “polluted” and “reference” (Fig. 1). The other statistical method, presented by Goltsev *et al.* [33] is neutral network. We have found the PCA analysis as a convenient tool in plant grouping.

Since the first PCA variable was dependent on measured parameters (Fig. 1) and also the points of inflection J and I were detectable on the logarithmic time scale (Fig. 2a) it was decided to use double normalized induction curves to compare the dynamics of electron transfer processes in various objects and thereby to estimate, which variety tolerates stresses better [10], [34], [35].

Analysis of normalized curves distinguish cv. Rahela. as the only outstanding cultivar with the shape of particular curves, different on all drown figures. The inflection point K on the fluorescence curve is associated with water photolysis and the amount of electron transported along Electron Transfer Chain (ETC); high relative fluorescence means slowing of electron flow from the chlorophyll Reaction Centers (RC) of Photosystem II (PSII) as a result of lower activity of PS II Oxygen Evolving Complex (OEC) and slower water splitting [36], [37]. The negative peak on ΔW_OJ_ curve, calculated as the difference between fluorescence of plants grown on the polluted area minus values of plants grown on the reference site, means that the relative slowing of the electron flow in chloroplasts of plants from polluted area was smaller than in chloroplasts from plants grown on the reference area. It can be hypothesized that in such a situation the ascorbate can be the electron donor in chloroplasts of plants from the polluted area [36], [37], [38], [39]. Again the cv. Rahela stood out, as on the Fig. 2b. Normalization at points O and K visualized L-band (Fig. 2d). A negative L-band indicates higher grouping of PSII antennas as compared to reference sample [35], [38], [40], which is the fact for all cultivars, beside cv. Rahela. The similarities of the shape of all normalized curves for all cultivars beside cv. Rahela may suggest the different physiological response to stress of that one. The y-axis shift is detected on all graphs. Cv. Wiwena. is at the bottom of Fig. 2b, Fig. 2c and Fig. 2d while cv. Rahela is at the top. Obtained results can be interpreted as a mark of cultivars reaction on HM ion stress. It looks that all cultivars except cv. Rahela. took from the soil only such amount of HM, which is not too stressful for them. Those cultivars, under the influence of HM ions stress accelerated metabolism, which is a typical physiological response of plants slightly resistant or non-resistant to the stress. Resistant plants typically launch additional defense mechanisms and rebuild its metabolism [41], [42]. Contrary to expectation, cv. Rahela which sequestrated the highest concentration of HM ions in the aerial part, did not show PSII antennas grouping under the stress, as other cultivars did. It should be considered that such cultivars which survive the stress by accelerating photosynthesis as well as the whole metabolism could be less resistant in front of additional environmental stresses such as i.e. drought or cold and could react more rapidly on them. *Festuca arundinacea* cv. Rahela in fact deals the best with the HM stress. Its antennas are not damaged, might be due to redox guard in the form of ascorbate which in other cultivars were not induced strongly enough to be visible on double normalized graphs.

## Conclusions

1. Chl *a* fluorescence is a good tool in phytoremediation studies.
2. The combined effects of elevated concentration of lead, cadmium and zinc in soil was manifested in alteration of some parameters of Chl *a* fluorescence as well as plant growth.
3. Heavy metal soil contamination resulted in biomass yield reduction of cultivars of *Bromus inermis* ‘Brudzynska’ and *B. carinathus* ‘Broma’.
4. *Festuca arundinacea* ‘Rahela’, may absorb relatively high amount of HM ions from the soil, without significant reduction of biomass yield, what is expected in the case of bioremediation practices. Mentioned cultivar could be regarded as a good species for the phytoextraction of Cd-contaminated soil.
